# The effect of intensive nutrition interventions on weight gain after kidney transplantation: protocol of a randomised controlled trial

**DOI:** 10.1186/1471-2369-15-148

**Published:** 2014-09-09

**Authors:** Kristin J Ryan, Jessie M Segedin Casas, Laura E Mash, Sandra L McLellan, Lyn E Lloyd, James W Stinear, Lindsay D Plank, Michael G Collins

**Affiliations:** Discipline of Nutrition, Faculty of Medical and Health Sciences, University of Auckland, Auckland, New Zealand; Nutrition Services, Auckland City Hospital, Auckland District Health Board, Auckland, New Zealand; Department of Sport and Exercise Science, Faculty of Science, University of Auckland, Auckland, New Zealand; Department of Surgery, Faculty of Medical and Health Sciences, University of Auckland, Auckland, New Zealand; Department of Renal Medicine, Auckland City Hospital, Auckland District Health Board, Private Bag 92024, Auckland, 1142 New Zealand; Department of Medicine, Faculty of Medical and Health Sciences, University of Auckland, Auckland, New Zealand

**Keywords:** Kidney transplantation, Weight gain, Obesity, Body composition, Nutrition intervention, Randomised-controlled trial

## Abstract

**Background:**

Weight gain and obesity are common after kidney transplantation, particularly during the first year. Obesity is a risk factor for the development of new-onset diabetes after transplantation, and is associated with reduced graft survival. There is a lack of evidence for effective interventions to prevent weight gain after kidney transplantation.

**Methods/Design:**

The effect of **INTE**nsive **N**utrition interventions on weight gain after kidney **T**ransplantation (INTENT) trial is a single-blind (outcomes assessor), randomised controlled trial to assess the effect of intensive nutrition interventions, including exercise advice, on weight gain and metabolic parameters in the first year after transplantation. Participants will be randomised during the first post-transplant month to either standard care (four visits with a renal dietitian over twelve months) or intensive nutrition intervention (eight visits with a renal dietitian over the first six months, four visits over the second six months, and three visits over the first six months with an exercise physiologist). In the intensive intervention group, nutrition counselling will be provided using motivational interviewing techniques to encourage quality engagement. Collaborative goal setting will be used to develop personalised nutrition care plans. Individualised advice regarding physical activity will be provided by an exercise physiologist. The primary outcome of the study is weight at six months after transplant, adjusted for baseline (one month post-transplant) weight, obesity and gender. Secondary outcomes will include changes in weight and other anthropometric measures over 12 months, body composition (in vivo neutron activation analysis, total body potassium, dual-energy X-ray absorptiometry, and bioelectrical impedance), biochemistry (fasting glucose, lipids, haemoglobin A1c and insulin), dietary intake and nutritional status, quality of life, and physical function.

**Discussion:**

There are currently few randomised clinical trials of nutrition interventions after kidney transplantation. The INTENT trial will thus provide important data on the effect of intensive nutrition interventions on weight gain after transplant and the associated metabolic consequences. Additionally, by assessing changes in glucose metabolism, the study will also provide data on the feasibility of undertaking larger multi-centre trials of nutrition interventions to reduce the incidence or severity of diabetes after transplantation.

**Trial registration:**

Australian New Zealand Clinical Trials Registry Number: ACTRN12614000155695

## Background

Kidney transplantation represents the optimal form of renal replacement therapy and, compared with dialysis treatment, is associated with significantly improved survival [1, 2], quality of life [3] and reduced costs over time [4, 5]. However, despite improvements in both patient and graft survival, kidney transplant recipients continue to have increased mortality compared with the general population [6]. This is predominantly due to an increased risk of cardiovascular disease [7, 8]. Excessive weight gain, obesity and diabetes are important risk factors for cardiovascular disease in transplant recipients, and are associated in multiple studies with an increased risk of death, cardiac events and graft loss (necessitating a return to dialysis treatment), even when confounding factors are taken into account [9–13].

In the first year following kidney transplantation, there is a significant incidence of excess weight gain and obesity in transplant recipients, with average post-transplant weight gains of 10-35% reported [14–18]. These changes are associated with increased total body fat, reduced lean muscle mass and reduced bone mineral density [17, 19–22]. Excess weight gain in the first year after transplant has also been shown to be a risk factor for the development of metabolic syndrome and new-onset diabetes after transplant (NODAT) [23–27]. Possible causes for weight gain include the use of immunosuppressive medications (such as corticosteroids), the relaxation of dietary restrictions associated with dialysis treatment (increased freedom of food and fluid choices, which may lead to increased energy intake), and improvements in well-being and appetite associated with the resolution of the uraemic state [10, 28–30]. In addition, studies indicate that many transplant recipients do not engage in adequate levels of physical exercise and activity, despite the improvements in overall well-being [31–33].

Despite concern about weight gain and obesity following kidney transplantation, not all post-transplant weight gain is considered undesirable. A high incidence of malnutrition has been reported in patients receiving dialysis therapies [34], even in those with a body mass index (BMI) in the overweight or obese range [35]. Low BMI, malnutrition and low muscle mass have been associated with poor survival after kidney transplantation [36, 37]. It is therefore important to consider not only weight but other markers of nutrition (including evidence for protein-energy wasting and serum albumin) and body composition in the assessment and treatment of kidney transplant recipients.

Importantly, weight gain in the first post-transplant year has been shown to be associated with reduced graft survival [38], and obese individuals have an increased risk of death and graft failure in the first 12 months compared with individuals of normal weight [39]. Weight gain after transplant therefore represents a potentially modifiable risk factor for poor outcomes after transplantation that might be an appropriate target for therapeutic intervention.

There are currently no evidence-based clinical guidelines on specific interventions to prevent or manage excessive weight gain and obesity after transplantation, due to a lack of high quality evidence from randomised controlled trials. On the basis of several non-randomised studies, the Caring for Australasians with Renal Impairment (CARI) guidelines have suggested that kidney transplant recipients be referred to a dietitian for advice to prevent weight gain post-transplant, and that this advice should be reinforced via follow-up assessments on a regular basis [40, 41]. Guidelines from the Kidney Disease: Improving Global Outcomes (KDIGO) initiative and the United Kingdom (UK) Renal Association have suggested that obesity is assessed at each visit and weight management services should be available for patients [42, 43].

In the general population, systematic reviews of randomised studies have shown that interventions addressing nutrition, behaviour and physical activity can be effective in reducing weight in obese patients, although the effects are often not sustained over the long term following the intervention programme [44–46]. Interventions that involve regular review (such as fortnightly during the first three months and continued monitoring for at least 12 months) have shown the greatest benefits [47, 48].

In kidney transplant patients, the few studies that have considered the effects of nutrition and exercise interventions on bodyweight within the 12 months after transplant have had inconsistent results: significantly less weight gain [49], a significant reduction in weight gain from baseline for males [50], or no effect on bodyweight [51]. However, the interpretation of results is limited by the study designs; two studies were non-randomised [49, 50] and one was designed to assess the effects of the intervention on hyperlipidaemia [51].

Overall, there is a need to develop effective interventions to minimise the detrimental effects of weight gain and obesity on long-term kidney transplant outcomes. Randomised controlled trials are needed to assess whether nutrition interventions are likely to be effective in preventing weight gain after kidney transplantation.

The effect of **INTE**nsive **N**utrition interventions on weight gain after kidney **T**ransplantation (INTENT) trial is a randomised controlled trial designed to evaluate the effect of an intensive nutrition intervention, including tailored advice regarding exercise and physical activity, on weight gain in the first year following kidney transplantation. In addition, detailed measures of the effects of the nutrition intervention on other anthropometric measures, body composition, glucose metabolism and biochemical markers of metabolic risk, functional capacity and quality of life will be performed. The trial will also provide data on the feasibility of conducting larger trials of nutrition interventions to reduce the incidence or severity of diabetes after kidney transplantation.

## Methods/Design

### Study aims and hypothesis

The primary aim of the INTENT trial is to determine whether the use of an intensive nutrition intervention, including physical activity advice, following kidney transplantation will be effective at reducing weight gain in the first year post-transplant.

Secondary aims are to determine whether the use of an intensive nutrition intervention will be associated with improvements in anthropometry, body composition, glucose metabolism and markers of metabolic risk; also, whether the use of an intensive nutrition intervention has any effects on dietary intake, nutritional status, quality of life, and physical function.

Our hypothesis is that early intensive nutrition intervention, including advice regarding physical activity, after kidney transplantation will be effective at reducing weight gain in the first post-transplant year, compared with standard post-transplant care.

The INTENT trial will be used to assess the feasibility of undertaking a larger study to determine if nutrition interventions early after kidney transplant can reduce the incidence and severity of diabetes after transplantation (and thus potentially the risk of cardiovascular events). The primary feasibility outcome will be whether a difference in haemoglobin A1c (HbA1c) is observed between the intervention and standard care groups at 12 months.

### Study design

**Figure 1:**
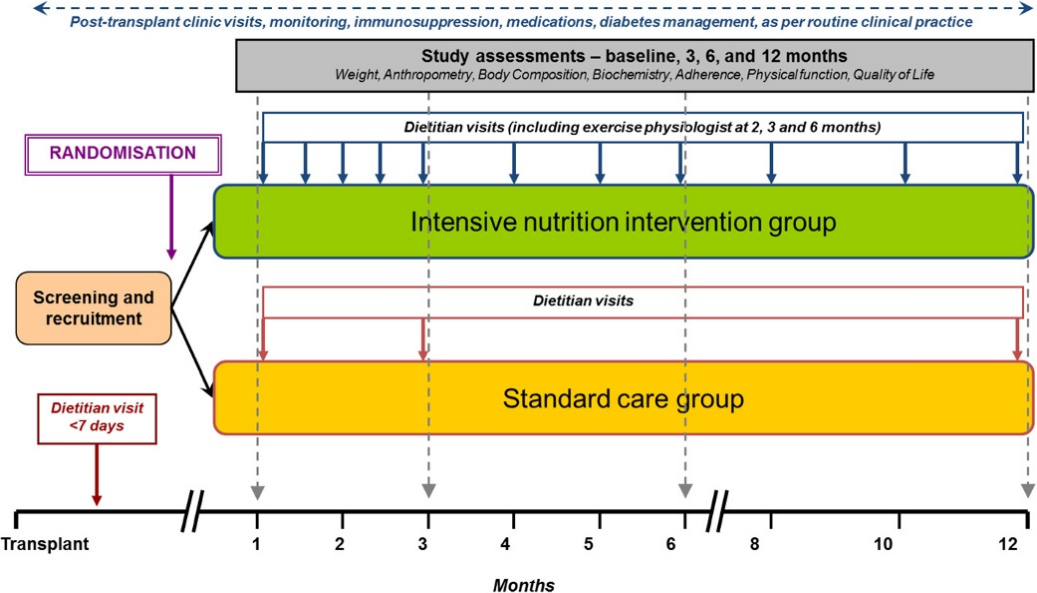
**Flowchart of the INTENT trial.**

The INTENT trial is a single-centre, single-blind, randomised controlled trial with outcomes assessment blinded to group allocations. An outline of the study is shown in Figure 1.

### Ethical considerations

Ethical approval has been obtained through the Northern B Health and Disability Ethics Committee of the Ministry of Health in New Zealand.

### Target population and eligibility criteria

The target population for the trial will be adult kidney transplant recipients, residing within the Auckland regional area, and recruited during routine post-transplant care within one month of a kidney transplant procedure at Auckland City Hospital.

Inclusion criteria will include the following: age > 18 years; stable graft function, as determined by the patient’s treating physician; willing and able to participate in all trial procedures for the duration of trial follow-up; and ability to provide written informed consent.

Exclusion criteria will include the following: severe obesity, defined by BMI >40 kg/m^2^; underweight, defined by BMI <18.5 kg/m^2^; evidence of significant malnutrition requiring enteral or parenteral nutrition therapy; ongoing significant medical complications that in the opinion of the patient’s treating physician preclude involvement in the trial; and considered likely to become pregnant during the duration of the trial.

Diabetes, cardiovascular disease or other stable medical co-morbidities post-transplant will not be exclusions *per se* unless deemed to represent a contraindication to participating in the trial by the patient’s treating physician. Patients who have been or are receiving treatment for acute rejection will not be excluded, providing graft function is stable at the time of entry to the trial in the opinion of the patient’s treating physician. Participation in another clinical trial will not be an exclusion providing that such participation will not compromise involvement in this trial, in the opinion of the principal investigator.

### Recruitment, randomisation and blinding

Potential participants will be identified during their initial inpatient stay following transplantation at Auckland City Hospital, or through the renal transplant outpatient clinic following discharge. Eligible transplant recipients will be invited to participate, given detailed information about the trial and, if agreeable, will be asked to provide written informed consent. In order to participate, transplant recipients will need to provide informed consent and be randomised prior to one month post kidney transplantation.

Participants will be randomised in a 1:1 ratio to receive either intensive nutrition intervention or standard of care. Randomisation will be performed externally by a statistical consultant using a computer-generated sequence allocation with permuted blocks of variable size, and stratified by gender. Allocation concealment will be via sealed opaque envelopes.

Formal study assessments of weight, anthropometry, measures of body composition, and physical function will be performed by one of the investigators, who will be blinded to the group allocation of participants. Laboratory staff blinded to the group allocations will perform all laboratory testing.

### Nature of nutrition care

Nutrition care will follow the Nutrition Care Process developed by the Academy of Nutrition and Dietetics, Chicago USA. This involves assessment, diagnosis of nutrition problem, intervention, monitoring and evaluation [52]. Australian and New Zealand evidence-based guidelines for the nutritional management of kidney transplant recipients will be used [53, 54].

### Standard care group

Participants randomised to the standard care group will receive standard post-transplant care as per usual local practice. This includes immunosuppressive medications to prevent rejection and other treatments under the direction of the treating physician and renal transplant team. Standard immunosuppression includes a calcineurin inhibitor (cyclosporine or tacrolimus as determined by the treating physician), mycophenolate, steroids, and basiliximab as induction therapy.

Current standard nutrition care after kidney transplantation at Auckland City Hospital includes assessment by a renal dietitian during the inpatient stay. Patients are given resources guiding them on healthy eating and food safety after transplant, and nutrition interventions to address any identified nutritional problems. Standard outpatient nutrition care involves an individual consultation with a specialist renal dietitian at 1, 3 and 12 months post-transplant alongside regular physician appointments.

### Intensive nutrition intervention group

Patients randomised to the intensive nutrition intervention group will, in addition to standard care, receive more frequent counselling with a specialist renal dietitian throughout the trial. These will commence at one month post-transplant and include an additional 8 visits overall compared to the standard care group (see Table 1). Participants will also receive individualised assessment and advice regarding physical activity and exercise, via formal consultations with an exercise physiologist at 2 months, 3 months and 6 months post-transplant.

**Table 1:** **Schedule of nutrition assessment**, **education and advice provided by renal dietitian and exercise physiologist** (**intensive nutrition intervention group**)

**Months 1** **-** **3**	**Fortnightly** review with dietitian to undertake initial assessments, determine progress, goals and adherence to recommendations
	Full review of dietary intake and adequacy of adherence to recommendations to be undertaken at 1 month and 3 months (including 3-day food diary and analysis)
	Exercise advice (including a consultation with an exercise physiologist at 8 and 12 weeks to provide tailored advice) regarding physical activity and a review of physical activity adherence
**Months 4** **-** **6**	**Monthly** review with dietitian to assess progress, goals and adherence to recommendations, and give further education and advice as appropriate
	Full review of dietary intake and adequacy of adherence to recommendations to be undertaken at 6 months (including 3-day food diary and analysis)
	Exercise advice (including a consultation with an exercise physiologist at 6 months to provide tailored advice) regarding physical activity and a review of physical activity adherence
**Months 7** **-** **12**	**Bi** **-** **monthly** review with dietitian to assess progress, goals and adherence to recommendations, and give further education and advice as appropriate
	A final review of diet history and adequacy of adherence to recommendations to be undertaken at 12 months (including 3-day food diary and analysis)
	Exercise advice to promote adequate physical activity and review of physical activity adherence

Individualised nutrition counselling will be provided using motivational interviewing techniques to encourage quality engagement. Personal nutrition plans will be developed using S.M.A.R.T. action steps, i.e. specific, measureable, achievable, relevant and time-limited [55].

Prior to full reviews at 1, 3, 6 and 12 months, a self-administered 3-day food diary record will be completed by participants and analysed using appropriate analysis software (e.g. FoodWorks® Professional [Xyris software]). Dietary intake data will be compared to evidence-based recommendations on nutrient requirements after renal transplant and used to guide nutrition prescriptions. At follow-up reviews, renal dietitians will assess progress, goal setting and adherence to dietary recommendations. Adherence will be measured using rulers to assess motivation to change, level of achievement of patient-centred goals and review of action steps taken in patient action plans.

### Primary and secondary outcomes

The primary outcome will be body weight at 6 months post-transplant, adjusted for weight at baseline (one month post-transplant), pre-existing obesity (BMI ≥30 kg/m^2^), and gender.

Secondary outcomes will include the following: change in weight and other anthropometric measures at 3, 6 and 12 months post-transplant; change in gold-standard body composition parameters post-transplant; validity of bio-electrical impedance assessment as compared with gold-standard body composition analysis in kidney transplant recipients; change in biochemical measures post-transplant (e.g. lipid profile and markers of insulin resistance); adherence to dietary advice in the intervention group; level of physical activity and physical functional capacity in transplant recipients; and quality of life in transplant recipients. In addition, a cost-effectiveness analysis of intensive nutrition intervention will be undertaken taking into account the costs of additional dietitian input (including missed visits), changes in weight and quality of life.

In order to determine the feasibility of undertaking larger multi-centre trials of nutrition interventions to reduce the incidence or severity of diabetes after transplantation, the difference in HbA1c observed between the intervention and standard care groups at 12 months will be reported as an exploratory feasibility outcome.

### Data collection

Standard demographic, clinical and laboratory data (including medication details) will be collected at baseline (1 month) and at 3, 6 and 12 months post-transplant. This will include details of any acute medical issues, clinical or other adverse events, graft function, acute rejection, hospital admissions, development of diabetes, or any other issues that might affect nutritional status or change in weight. Formal study assessments (blinded to group allocation) will be undertaken at baseline (1 month), and at 3, 6 and 12 months (Table 2).

**Table 2:** **INTENT study data collection at baseline and months 3**, **6 and 12 post**-**transplant in the intervention and standard care groups**

Type of data	Specific measure
**Demographics**	Age
	Gender
**Clinical status**	Acute medical issues (including acute rejection, hospital admissions, diabetes status)
	Graft function
**Anthropometry**	Body weight
	Body mass index
	Waist circumference & waist-hip ratio
	Mid-arm circumference
	Skin-fold thickness
**Body composition**	Bioimpedance assessment
	Deuterium dilution (total body water, fat mass)
	DEXA (bone mass and fat mass)
	*In vivo* neutron activation analysis of body nitrogen (total body protein)
	Total body potassium (body cell mass)
**Biochemistry**	Serum creatinine
	Estimated GFR
	Immunosuppression drug levels
	Fasting glucose
	Insulin
	HbA1c
	Total, LDL & HDL cholesterol and triglycerides
	Insulin resistance (HOMA)
Dietary*	3-day food diary
	Motivational assessment rulers to assess motivation to change
**Physical function**	Gait speed assessment
	Hand grip strength
	Sit-to-stand-to-sit test
**Other**	PG-SGA
	NZPAQ-SF
	SF-36
	Blood pressure

*Only collected for intensive arm.

DEXA = Dual-energy x-ray absorptiometry; GFR = Glomerular filtration rate; HbA1c = Haemoglobin A1c; HDL = High density lipoprotein; LDL = Low density lipoprotein; NZPAQ-SF = New Zealand physical activity questionnaire – short form; PG-SGA = Patient-generated subjective global assessment; SF-36 = Short Form-36.

### Anthropometry and body composition

Standardised anthropometric measurements will include body weight, BMI, waist circumference and waist-hip ratio, mid-arm circumference and skin-fold thickness. These will be performed using calibrated instruments stored permanently in the Body Composition Laboratory at the University of Auckland. To ensure standardisation, the same equipment will be used for all measurements in all patients.

Body composition measurements will include gold-standard methods: dual-energy x-ray absorptiometry (DEXA) to determine fat mass [56, 57], total body potassium to assess body cell mass [58], *in vivo* neutron activation analysis for body nitrogen to determine total body protein [59], and deuterium dilution to determine total body water [60]. In addition, bioelectrical impedance assessment will be carried out as a surrogate measurement of total body water, extracellular water and fat mass [61].

### Nutritional status, physical activity and quality of life

Nutritional status will be assessed using the patient-generated subjective global assessment (PG-SGA) [62, 63]. Physical activity will be assessed using the New Zealand Physical Activity Questionnaire – short form (NZPAQ-SF), a modified version of the International Physical Activity Questionnaire (IPAQ) [64, 65]. The Short Form 36 (SF-36) will be used to assess quality of life [66].

### Physical function

The following physical function assessments will be carried out: gait speed, hand grip strength, and sit-to-stand-to-sit test (to assess lower extremity strength) [67–69].

### Biochemistry

During the formal study assessments at baseline (1 month), 3, 6 and 12 months, specific biochemical markers of glucose metabolism and metabolic risk will be measured. Blood samples will be collected after an overnight fast and assayed for glucose, insulin, HbA1c, lipids (including total cholesterol, low density lipoprotein [LDL], high density lipoprotein [HDL] cholesterol and triglycerides) [70]. The homeostatic model assessment (HOMA) index of insulin resistance will be calculated. In addition, where consent is given, serum samples will be stored for the analysis of novel risk factors in the future. Participants and investigators will be blinded to the serum outcomes.

In addition, standard tests will be taken as part of routine post-transplant care, including full blood count, serum creatinine, urea and electrolytes, other biochemistry and samples for therapeutic drug monitoring of tacrolimus or cyclosporine. The results of standard testing will not be blinded.

### Statistical analysis

The primary outcome (body weight at 6 months post-transplant) will be compared between the groups using an analysis of covariance (ANCOVA) adjusted for baseline weight, baseline obesity (BMI ≥ 30 kg/m^2^), and gender. The extent of weight gain after transplant has been shown be significantly affected by baseline BMI [18]; gender will be included as a covariate due to its use as a stratification variable in the randomisation.

Secondary analyses will include analyses of change in anthropometry (BMI, waist circumference, and waist-hip ratio), physical function, body composition, quality of life, and biochemistry parameters between the groups, using ANCOVA with adjustment for relevant baseline covariates. Repeated measures analyses using linear mixed models with multiple covariates will be used to compare changes in weight, anthropometry, physical function, body composition, quality of life, and biochemistry parameters over time (baseline, 3, 6 and 12 months) between the groups. Multiple linear regression will be used to identify significant predictors other than group allocation associated with these changes. Variables identified as significant will be analysed with pairwise comparisons.

Comparisons between groups for differences in variables of interest will be conducted using Fisher’s exact test for categorical variables, unpaired t tests for parametric variables, and Wilcoxon rank sum tests for non-parametric variables.

All statistical analyses will be performed using appropriate statistical software, such as STATA or SAS. The level of statistical significance will be set at probability level of <0.05.

### Calculation of required sample size

Using Stata statistical software to model an ANCOVA analysis of the primary outcome at six months, with power of 80% and alpha level of 5%, we estimate that 14 participants will be required per group, i.e. 28 in total. To account for dropouts, we aim to recruit up to 32 participants into the study. The effect size was estimated as a weight difference of 5 kg at 6 months between the groups, 74 kg (SD 9 kg) in the standard group and 69 kg (SD 12 kg) in the intervention group. These estimates reflect data from a non-randomised study of intensive nutrition interventions versus standard care in a comparable population of kidney transplant recipients whose weights were similar at baseline [49]. The correlation between weight at baseline and 6 months was estimated as 0.9, based on data showing a mean difference in weight of 3.2 kg (SD 5.9 kg) between 1 and 6 months of 1299 kidney transplant recipients who received their kidney transplant in Auckland between 1991 and 2012 (unpublished observations, data on file), where the correlation was 0.9514. Similar findings were found in an analysis of weight change post-transplant in a comparable population of 156 transplant recipients in Brisbane, Australia (mean weight change 3.3 kg, SD 6.0 kg) (Campbell KL, personal communication) [18].

## Discussion

This trial will provide evidence on the effects of intensive nutrition intervention on weight gain after kidney transplantation. In addition, the study will also examine in detail changes in other anthropometric indices (including BMI, waist circumference and waist-hip ratio), body composition, physical function, and biochemistry including glucose metabolism and markers of metabolic risk. We hypothesise that the intervention (compared to standard care) will result in reduced weight gain and lead to corresponding reductions in BMI, waist circumference and waist-hip ratio, improved physical function and favourable changes in body composition including reduced fat mass and increased muscle mass. We also hypothesise that these changes will be associated with improvements in glucose metabolism, reduced lipid levels and reduced insulin resistance.

The outcomes from this research will also provide important data on the feasibility of conducting larger multi-centre randomised trials of intensive nutrition interventions to reduce the incidence and severity of diabetes in kidney transplant recipients, and thus the potential to reduce the risks of cardiovascular disease in transplant recipients.

The use of the gold-standard body composition techniques including *in vivo* neutron activation analysis, deuterium dilution, total body potassium and DEXA, as well as the indirect but more readily available measures such as bioelectrical impedance, is a particular strength of this study. Few investigators have the ability to perform these gold-standard analyses and this study will provide important updated data on changes in these measures after kidney transplantation in the current era [71]. The use of these body composition measures will enable a much more detailed understanding of any changes in weight and anthropometry that are observed as a result of the intervention. We will also be able to compare the surrogate bioelectrical impedance measures that are more readily available with gold standard measures and thus provide important validation data in this population.

Historical data from a non-randomised study of nutrition interventions in the early period post-transplant [49] have been used to estimate the effect of the intervention on weight change after transplant. More recent data are not available to provide a more reliable estimate of the likely effect size, and it is thus possible that there may be more variability in weight gain observed in this population, and hence a larger sample size may be required for the same power. Some protection, however, is afforded by the fact that the primary outcome analysis will be accomplished using ANCOVA, rather than a t-test on weight change as used in the Patel study [49]. Previous data on post-transplant weight change in our population shows a high degree of correlation between weight at baseline (one month) and six months, supporting the use of an ANCOVA [72].

Due to the nature of the intervention, it is not possible to blind participants or investigators involved in the intervention to group allocations, and thus there is a potential risk of bias. We have attempted to minimise this by using single-blind design, i.e. by having formal measurements of weight and other measures that will be reported as study outcomes performed by a blinded investigator. In addition, to reduce the potential for confounding due to measurement variability, a single investigator will perform all of the outcome measurements using the same instruments throughout the study.

## Conclusions

The INTENT trial will provide important data on the effectiveness of nutrition interventions to prevent excessive weight gain after transplantation. Given the current lack of data from randomised controlled trials assessing the benefit of strategies to prevent excessive weight gain, this study therefore addresses an important gap in current evidence regarding optimal care after kidney transplantation.

## Abbreviations

ANCOVA: Analysis of covariance
BMI: Body mass index
CARI: Caring for Australasians with Renal Impairment
DEXA: Dual-energy x-ray absorptiometry
GAS: Goal attainment scaling
GFR: Glomerular filtration rate
HbA1c: Haemoglobin A1c
HDL: High density lipoprotein
HOMA: homeostatic model assessment
INTENT trial: The effect of **INTE**nsive **N**utrition interventions on weight gain after kidney **T**ransplantation trial
IPAQ: International physical activity questionnaire
KDIGO: Kidney Disease: Improving Global Outcomes
LDL: Low density lipoprotein
NODAT: New onset diabetes after transplant
NZPAQ-SF: New Zealand physical activity questionnaire – short form
PG-SGA: Patient-generated subjective global assessment
SF-36: Short Form-36.
